# Supplementary material for: Wide-field time-gated SPAD imager for phasor-based FLIM applications
Source: Methods Appl Fluoresc. Author manuscript; Available in PMC 2022 Feb 9. (PMC8827132; doi:10.1088/2050-6120/ab6ed7)
Supplement: SI [file NIHMS1772745-supplement-SI.pdf]

Supplementary Information

Supplementary information for “Wide-field time-gated SPAD imager for phasor-based FLIM applications” by Arin Ulku, Andrei Ardelean, Michel Antolovic, Shimon Weiss, Edoardo Charbon, Claudio Bruschini, Xavier Michalet

Content:

- Supplementary discussion
- Supplementary table
- Supplementary figures
- Supplementary references

SUPPLEMENTARY DISCUSSION

In this supplement, we provide the derivation of Eq. (11)-(13) in the main text.

Consider arrival times $\{t_i\}$ uniformly distributed in an interval [0, *W*].

Their average $<t_i>$ is given by:

$$\langle t_i \rangle = \frac{1}{W}\int_0^W t dt = \frac{1}{W}\left[\frac{t^2}{2}\right]_0^W = \frac{W}{2} \tag{1}$$

Similarly, the average of their squares is given by:

$$\langle t_i^2 \rangle = \frac{1}{W}\int_0^W t^2 dt = \frac{1}{W}\left[\frac{t^3}{3}\right]_0^W = \frac{W^2}{3} \tag{2}$$

Their variance is therefore given by:

$$\langle t_i^2 \rangle - \langle t_i \rangle^2 = \frac{W^2}{12} \tag{3}$$

which yields Eq. (10) of the main text.

We next compute the average of N such uniformly distributed arrival times:

$$\theta_N = \frac{1}{N}\sum_{i=1}^{N} t_i \tag{4}$$

The random variable (RV) θ_N defined as above is distributed according to the Bates distribution (1):

$$p(\theta;N) = \frac{N}{2(N-1)!}\sum_{k=0}^{N}(-1)^k \frac{N!}{k!(N-k)!}(N\theta-k)^{N-1}\sigma(N\theta-k) \tag{5}$$

where $\sigma(x)$ is the sign function ($\sigma(x) = -1$ if $x<0$, $\sigma(x) = 1$ if $x>0$, $\sigma(0) = 0$).

The mean and variance of the Bates distribution are given by:

$$\begin{cases} \langle \theta_N \rangle = \dfrac{W}{2} \\ \langle \theta_N^2 \rangle - \langle \theta_N \rangle^2 = \dfrac{W^2}{12N} \end{cases} \tag{6}$$

from which Eq. (11) of the main text follows.

In the case of photons corresponding to an exponentially decaying fluorescent source with lifetime τ, the arrival times $\{t_i\}$ detected by a gated detector with gate width W and gate boundaries $[\mu_j - W/2, \mu_j + W/2]$ can be decomposed into:

$$\begin{cases} t_i = \mu_{j_i} + \delta t_i \\ \delta t_i \in \left[-\frac{W}{2}, \frac{W}{2} \right] \end{cases} \tag{7}$$

where the gate center locations, μ_j, are exponentially distributed with mean and variance τ, and the $\delta\tau_i$ are uniformly distributed. θ_N, the RV defined above as the average of N such arrival times, can thus be written as the sum of two independent RVs:

$$\theta_N = \frac{1}{N} \sum_{i=1}^{N} \mu_{j_i} + \frac{1}{N} \sum_{i=1}^{N} \delta t_i \tag{8}$$

Because these two random variables are independent, their mean and variance add up:

$$\begin{cases} \langle \theta_N \rangle = \tau \\ \langle \theta_N^2 \rangle - \langle \theta_N \rangle^2 = \frac{\tau^2}{N} + \frac{W^2}{12N} \end{cases} \tag{9}$$

To obtain the above result, from which Eq. (13) of the main text follows, we have used the fact the first RV in Eq. (8) is the average of N exponentially distributed RV. The sum of N identical exponentially distributed RVs is distributed according to an Erlang distribution (2):

$$Erl(x; N, \tau) = \frac{}{(N-1)!\tau} \left(\frac{x}{\tau} \right)^{N-1} \exp\left(-\frac{x}{\tau} \right) \tag{10}$$

Its mean and variance are given by:

$$\begin{cases} \langle Erl(x; N, \tau) \rangle = N\tau \\ \operatorname{var}(Erl(x; N, \tau)) = N\tau^2 \end{cases} \tag{11}$$

from which the mean and variance of $1/N$ the same RV (which intervenes in Eq. (8)) follow.

SUPPLEMENTARY TABLE

TABLE S1: Parameters of 7 gate configurations tested for time-resolved imaging firmware of SwissSPAD2. The gate length W characterizes the exposure time per laser period (T = 50 ns in these experiments) and may differ slightly across the array (dispersion characterized by a full-width-at-half-maximum – FWHM) reported in ps. The theoretical square profile of the gate is in practice replaced by a plateau preceded by a finite rise time and followed by a fall time, both of which vary slightly across the array. This dispersion is characterized by a mean rise and fall times, as well as a rise and fall time FWHM reported in ps.

	**Gate Configuration**						
	**1**	**2**	**3**	**4**	**5**	**6**	**7**
**Gate Length (mean) [ns]**	10.8	11.7	13.1	15.5	17.9	20.3	22.7
**Gate Length (FWHM) [ps]**	306	256	226	166	139	120	112
**Rise Time (mean) [ps]**	320	383	378	333	371	339	375
**Fall Time (mean) [ps]**	618	616	617	617	622	617	615
**Rise Time (FWHM) [ps]**	60	65	70	64	56	57	62
**Fall Time (FWHM) [ps]**	80	79	79	79	79	79	79
**Rising Edge Position (FWHM) [ps]**	319	269	252	193	185	161	148
**Falling Edge Position (FWHM) [ps]**	219	219	221	216	218	220	218

SUPPLEMENTARY FIGURES

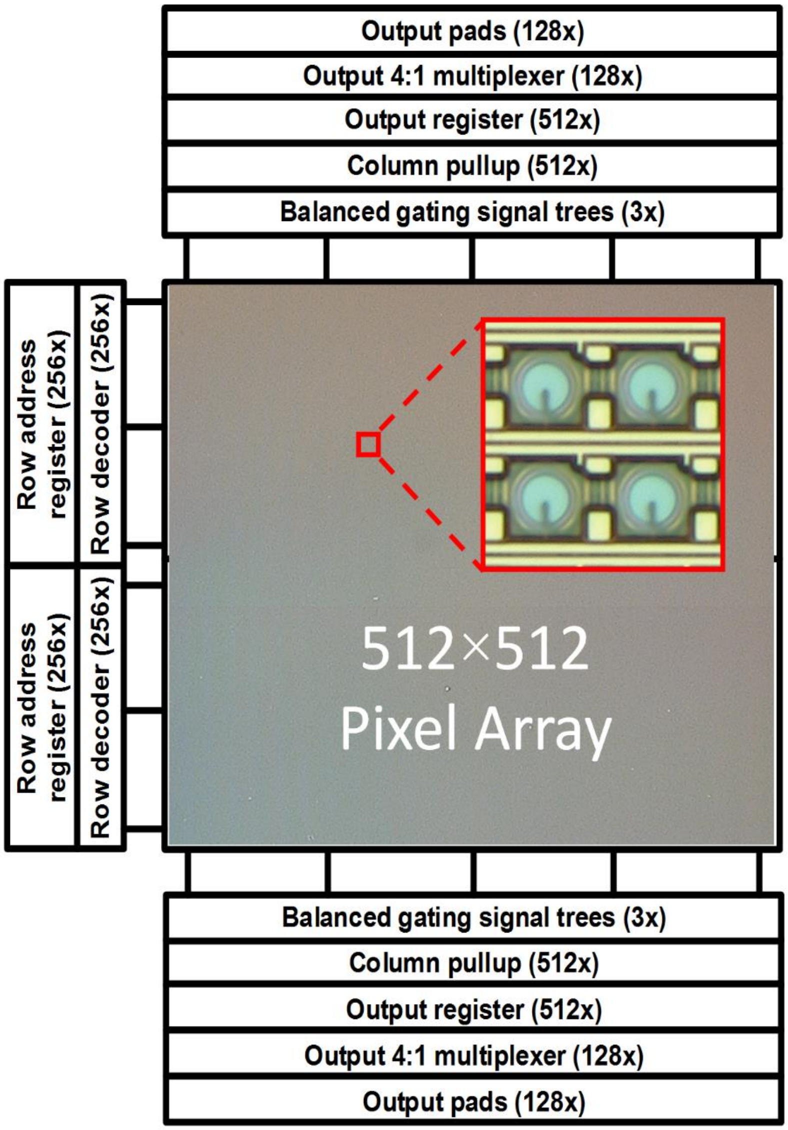

Fig. S1. Die micrograph and block diagram of the SwissSPAD2 image sensor (3).

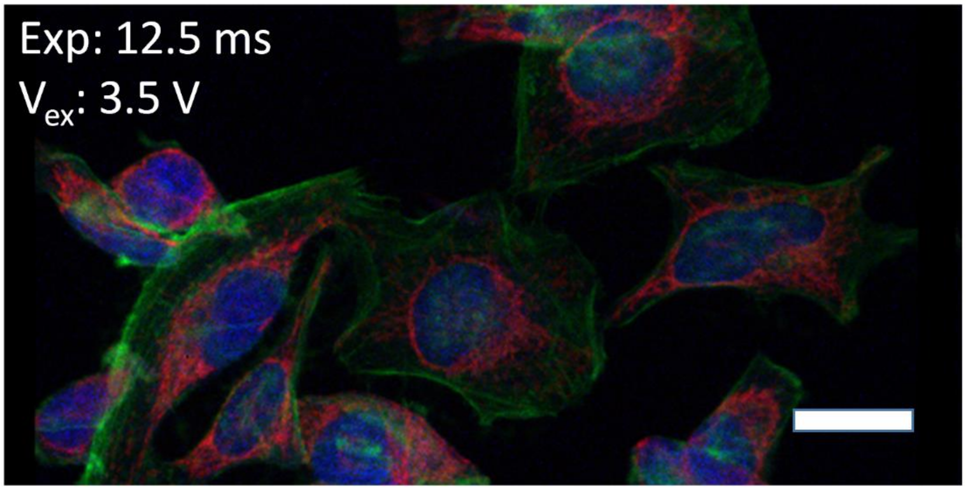

Fig. S2. A multicolor fluorescence intensity image of HeLa cells labeled with 3 different fluorescent dyes, captured by SwissSPAD2 (3). Scale bar is 26.2 μm.

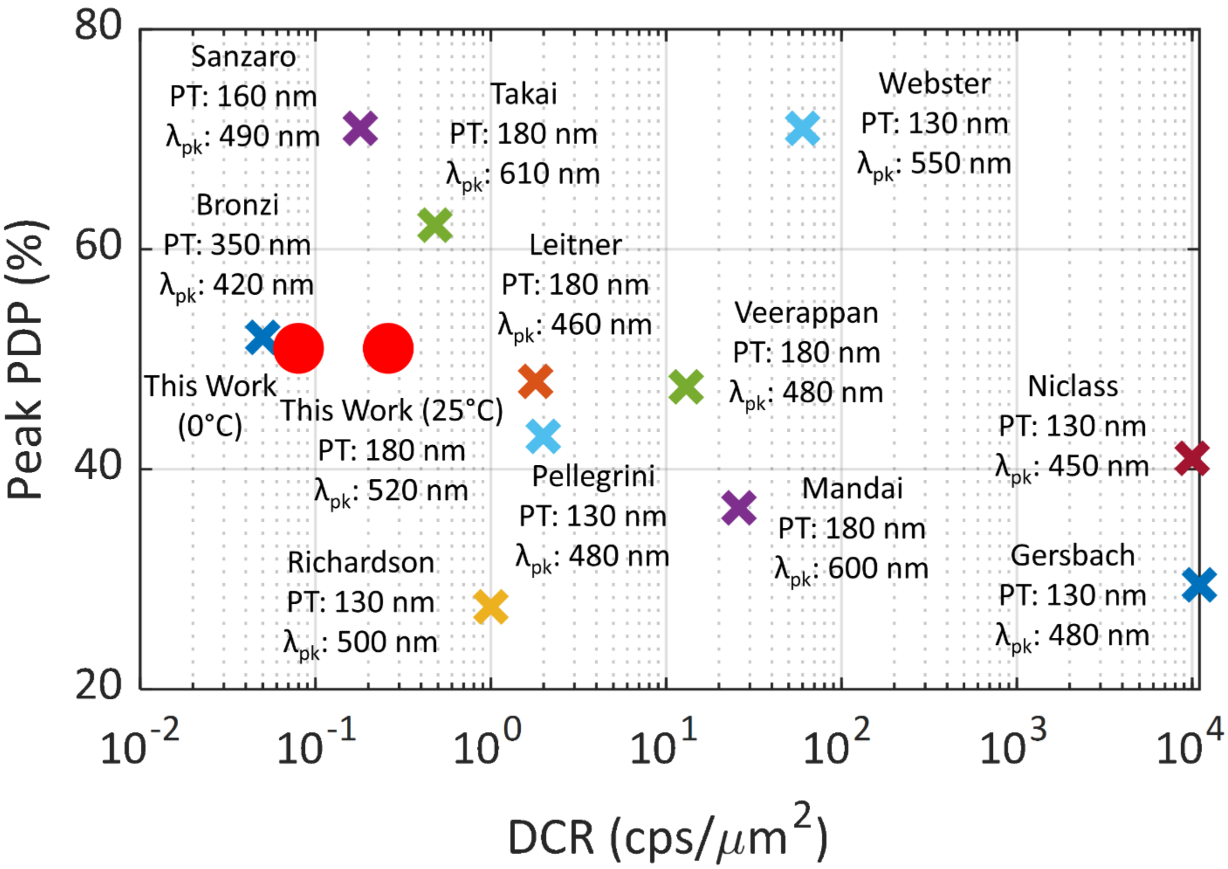

Fig. S3. PDP and DCR performance of SPADs fabricated in standard CMOS process technologies (3). (PT: Process technology, λ_{pk}: Peak wavelength). References cited : (4-14).

Sample 1

-0.001	0.007	0.007	0.006	-0.001
0.004	0.034	0.057	0.032	-0.003
0.000	0.056	100	0.059	0.001
0.001	0.030	0.053	0.029	0.003
-0.001	0.006	0.004	0.000	0.001

Sample 2

0.004	0.004	0.003	0.001	0.002
-0.001	0.030	0.059	0.031	0.007
0.012	0.056	100	0.073	0.001
0.007	0.032	0.056	0.031	0.003
-0.005	0.002	0.008	-0.001	0.000

Sample 3

0.002	0.000	0.006	0.000	0.002
0.005	0.028	0.054	0.029	0.005
0.003	0.061	100	0.064	0.009
0.000	0.032	0.071	0.032	-0.002
0.005	0.001	0.008	-0.003	0.002

Sample 4

-0.003	0.006	0.006	-0.001	0.000
-0.003	0.026	0.056	0.032	0.004
0.007	0.060	100	0.055	0.007
-0.001	0.026	0.055	0.029	0.002
-0.003	0.004	0.003	0.001	-0.004

Sample 5

0.006	0.001	0.005	0.006	-0.005
0.005	0.033	0.062	0.032	0.005
0.013	0.067	100	0.065	0.008
0.000	0.035	0.066	0.025	-0.002
0.009	0.003	0.003	0.002	0.003

Fig. S4. Crosstalk characterization results of five SwissSPAD2 samples at 6.5 V excess bias voltage. In each matrix, the values indicate the crosstalk probability of the pixels surrounding the reference pixel in the center. The values are calculated as the normalized median photon counts of multiple hot pixels and their neighbors in a dark image. The median dark count was subtracted from all pixels before the calculation. The results show that the crosstalk probability between nearest neighbors is less than 0.075%.

References

1. Bates GE 1955 Joint Distributions of Time Intervals for the Occurrence of Successive Accidents in a Generalized Polya Scheme *Ann Math Stat* **26** 705-720.
2. Krishnamoorthy K. 2006. Handbook of Statistical Distributions with Applications. Chapman & Hall/CRC.
3. Ulku AC, Bruschini C, Antolovic IM, Kuo Y, Ankri R, Weiss S, Michalet X, Charbon E 2019 A 512×512 SPAD Image Sensor with Integrated Gating for Widefield FLIM *IEEE J. Sel. Topics Quantum Electron.* **25** 6801212.
4. Takai I, Matsubara H, Soga M, Ohta M, Ogawa M, Yamashita T 2016 Single-Photon Avalanche Diode with Enhanced NIR-Sensitivity for Automotive LIDAR Systems *Sensors-Basel* **16** 459.
5. Leitner T *et al.* 2013 Measurements and Simulations of Low Dark Count Rate Single Photon Avalanche Diode Device in a Low Voltage 180-nm CMOS Image Sensor Technology *IEEE T. Electron. Dev.* **60** 1982-1988.
6. Veerappan C, Charbon E 2016 A Low Dark Count p-i-n Diode Based SPAD in CMOS Technology *IEEE T. Electron. Dev.* **63** 65-71.
7. Pellegrini S, Rae B 2017 Fully industrialised single photon avalanche diodes *Proc. SPIE* **10212** 102120D.
8. Gersbach M, Maruyama Y, Trimananda R, Fishburn MW, Stoppa D, Richardson JA, Walker R, Henderson R, Charbon E 2012 A Time-Resolved, Low-Noise Single-Photon Image Sensor Fabricated in Deep-Submicron CMOS Technology *IEEE J. Solid-St. Circ.* **47** 1394-1407.
9. Sanzaro M, Gattari P, Villa F, Tosi A, Croce G, Zappa F 2018 Single-Photon Avalanche Diodes in a 0.16 mu m BCD Technology With Sharp Timing Response and Red-Enhanced Sensitivity *Ieee J Sel Top Quant* **24**.
10. Webster EAG, Grant LA, Henderson RK 2012 A High-Performance Single-Photon Avalanche Diode in 130-nm CMOS Imaging Technology *Ieee Electr Device L* **33** 1589-1591.
11. Niclass C, Gersbach M, Henderson R, Grant L, Charbon E 2007 A single photon avalanche diode implemented in 130-nm CMOS technology *Ieee J Sel Top Quant* **13** 863-869.
12. Mandai S, Fishburn MW, Maruyama Y, Charbon E 2012 A wide spectral range single-photon avalanche diode fabricated in an advanced 180 nm CMOS technology *Opt. Express* **20** 5849-5857.
13. Richardson JA, Grant LA, Henderson RK 2009 Low Dark Count Single-Photon Avalanche Diode Structure Compatible With Standard Nanometer Scale CMOS Technology *Ieee Photonic Tech L* **21** 1020-1022.
14. Bronzi D *et al.* 2012. Low-noise and large-area CMOS SPADs with timing response free from slow tails. In 2012 Proceedings of the European Solid-State Device Research Conference (ESSDERC). 230-233.
